# Supplementary material for: Viroporins of Mpox Virus
Source: Int J Mol Sci. 2023 Sep 7;24(18):13828. doi: 10.3390/ijms241813828 (PMC10530900; doi:10.3390/ijms241813828)

Supplementary Table S1. Summary of the bacteria-based assay results for channel activity for the different viral proteins. The negative assay results represent the relative growth inhibition at 60 µM IPTG (Figure 1). The positive assay results are taken from Figure 2. The pH assay results are obtained from the slopes of linear regressions of the results presented in Figure 3.

Protein	Negative assay		Positive assay		pH assay		Average
	Value	Rank	Value	Rank	Value	Rank	Rank
C20.5L	63%	2	34%	5	-0.0051	7	5
A15.5L	45%	4	93%	2	-0.0238	2	1
gp124	-3%	8	-43%	8	-0.0324	1	6
gp063	23%	5	35%	4	-0.0222	3	3
gp066	11%	7	6%	6	-0.0125	6	8
gp125	21%	6	0%	7	-0.0152	4	6
gp081	56%	3	687%	1	-0.0149	5	2
gp120	68%	1	58%	3	-0.0028	8	3

Supplementary Figure S1. Sequences of the analyzed viral proteins and the putative transmembrane sequences according to TMHMM [30,31] are highlighted in yellow.

Proteins	Protein sequence with putative transmembrane domain(s)
C20.5L	MVIGLVIFVSVAATIVGVLSNVLDMIMYVEENNEEDAKIKEEQELLLLY
A15.5L	MISNYEPLLLLVITCCVLLFNFTISSKTKIDIIFAVQTIVFIWFIFHFVYSAI
gp124	MIGILLLIGICVAVTVTILYTLYNKIKNPQNPNPSPNLNSPPPETRNTKFVNNLEKDHISSLYNLVKSSA
gp063	MDKLYAAIFGVFMGSQEDDLTDFIEIVKSVLSDEKTVTSTNNTGCWGWYWLIIIFFIVLILLLLIYLYLKVVW
gp066	MVDAITVLTAICITVLMLLMVISGTAMIVKELNPNDIFTMQSLKFNRTVTIFKYIGLFIYIPGTIILYATYVKSLLMKN
gp125	MDMMLMIGNYFSGVLIAGIILLILSCIFAFIDFSKSTSPTRTWKVLSIMAFILGIIITVGMLIYSMWGKHCAPHRVSGVIHTNHSDISMN
gp081	MEVIADRLDDIVKQNIADEKFVDFVIHGLEHQCPAILRPLIRLFIDILLFVIVIYIFTVRLVSRNYQILLVLVALVITLTIFLLLYTIIVLD
gp120	MSCYTAILKSVGGLALFQVANGAIDLCRHFFMYFCEQKLRPNSFWFVVVRAIASMIMYLVLGIALLYISEQDDKKNTNNDSNSNNDKRNVSSINSNSSHK

Supplementary Figure S2. Protein structure prediction by AF2 [35] for the viral proteins from trimers to hexamers. The different panels depict the per-residue confidence estimates (pLDDT) obtained from AF2 of the top five ranked structures for each of the different oligomeric structures.

Supplementary Figure S2a.

C20.5L trimer

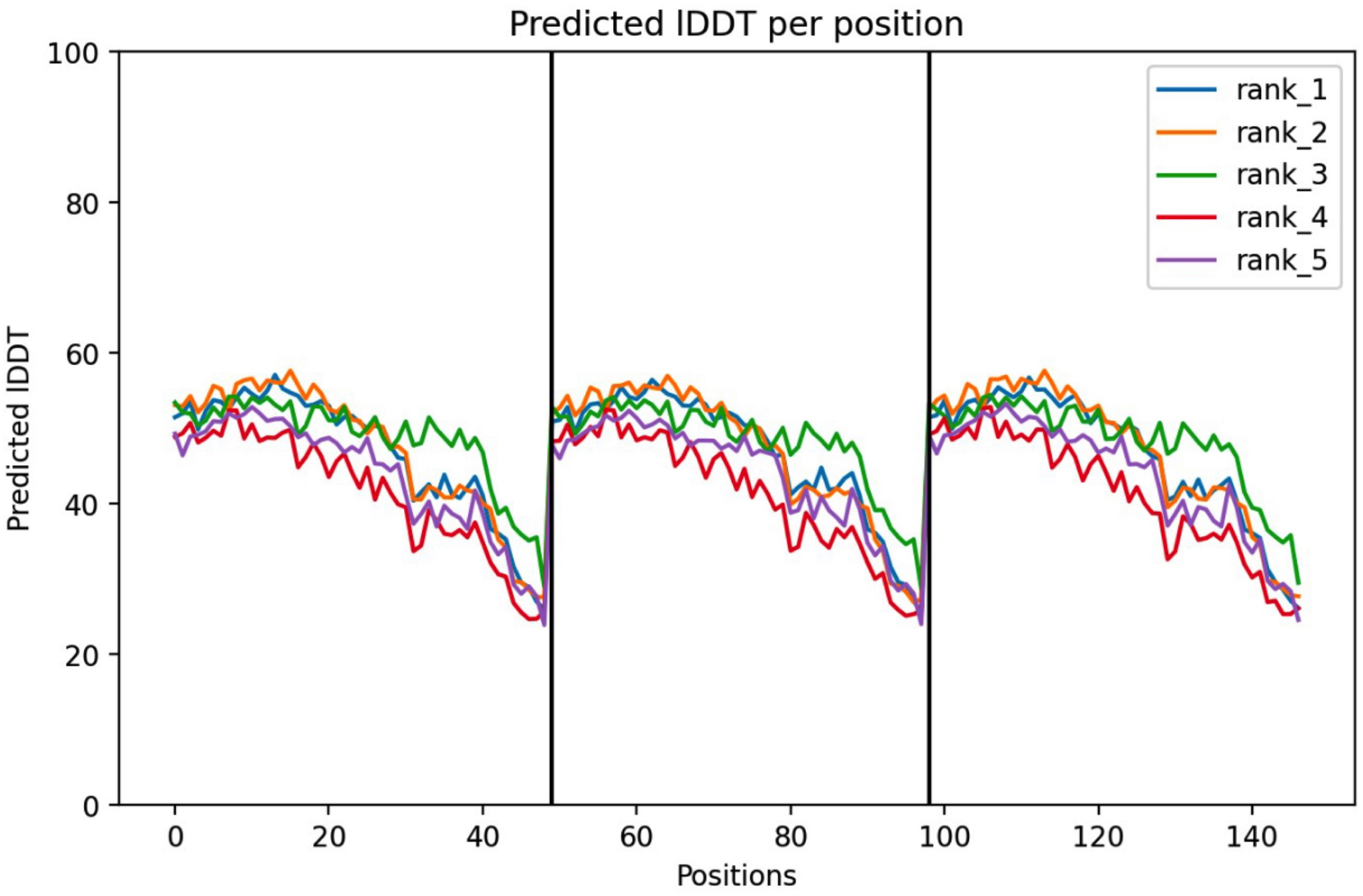

C20.5L tetramer

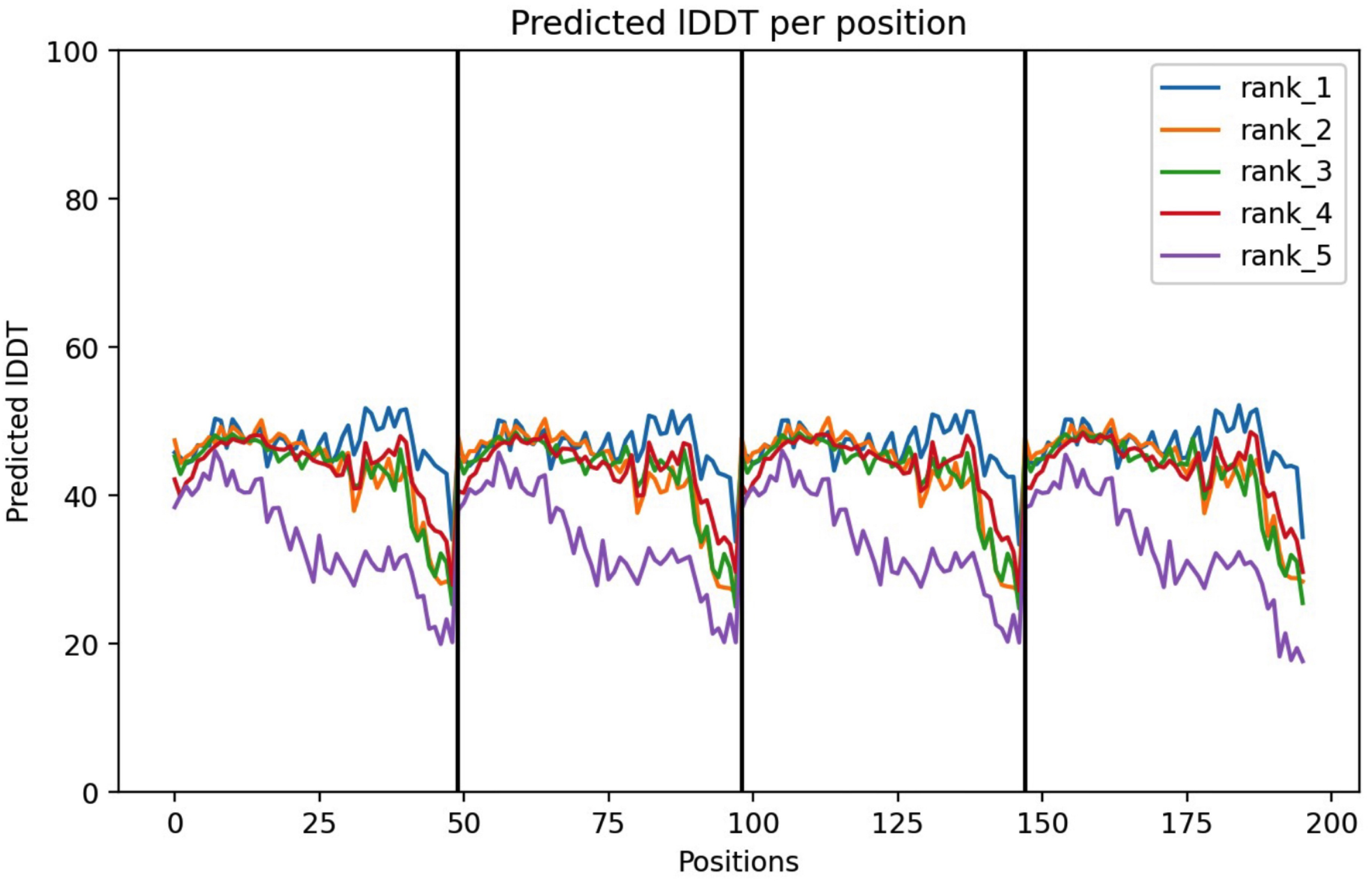

Supplementary Figure S2b.

C20.5L pentamer

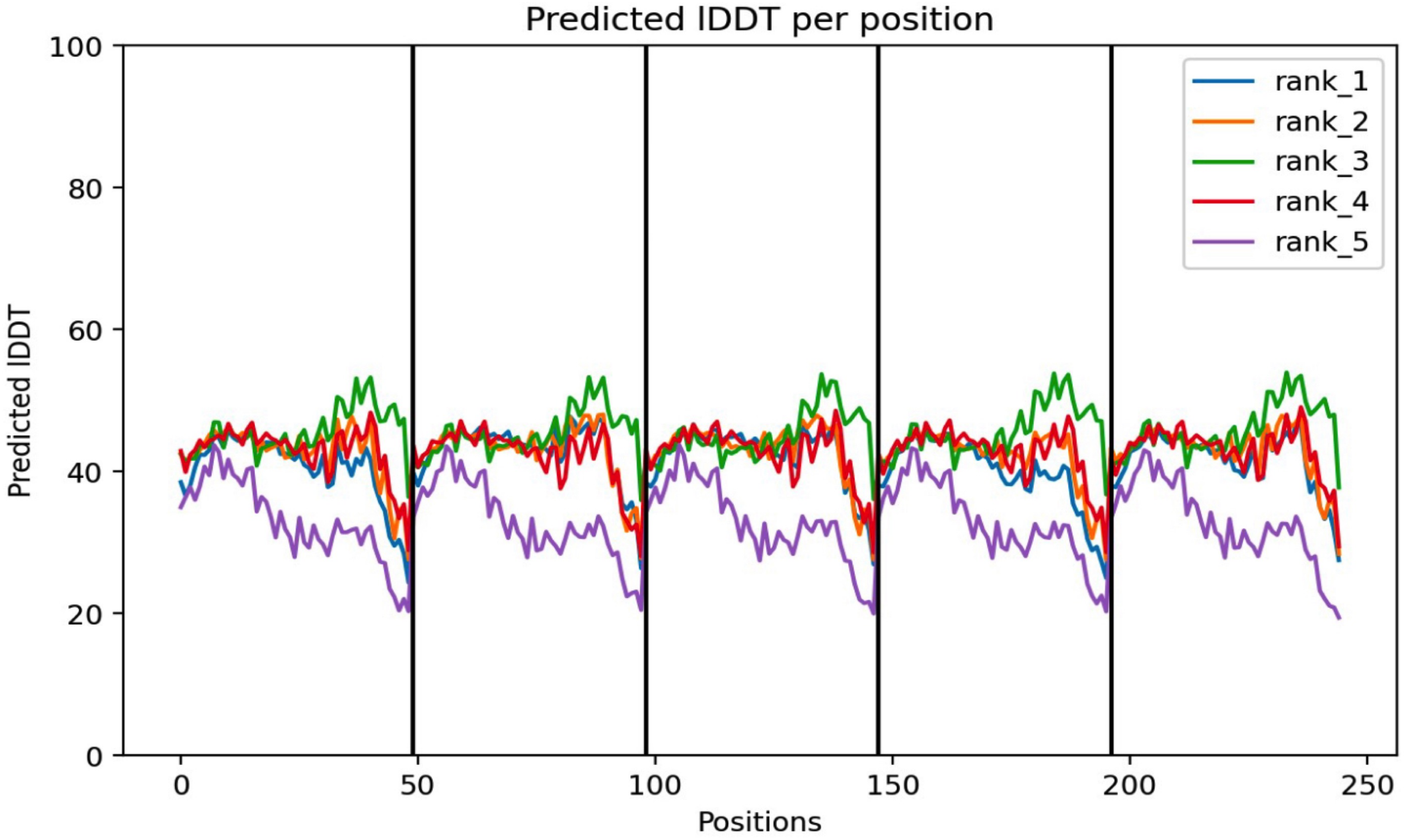

C20.5L hexamer

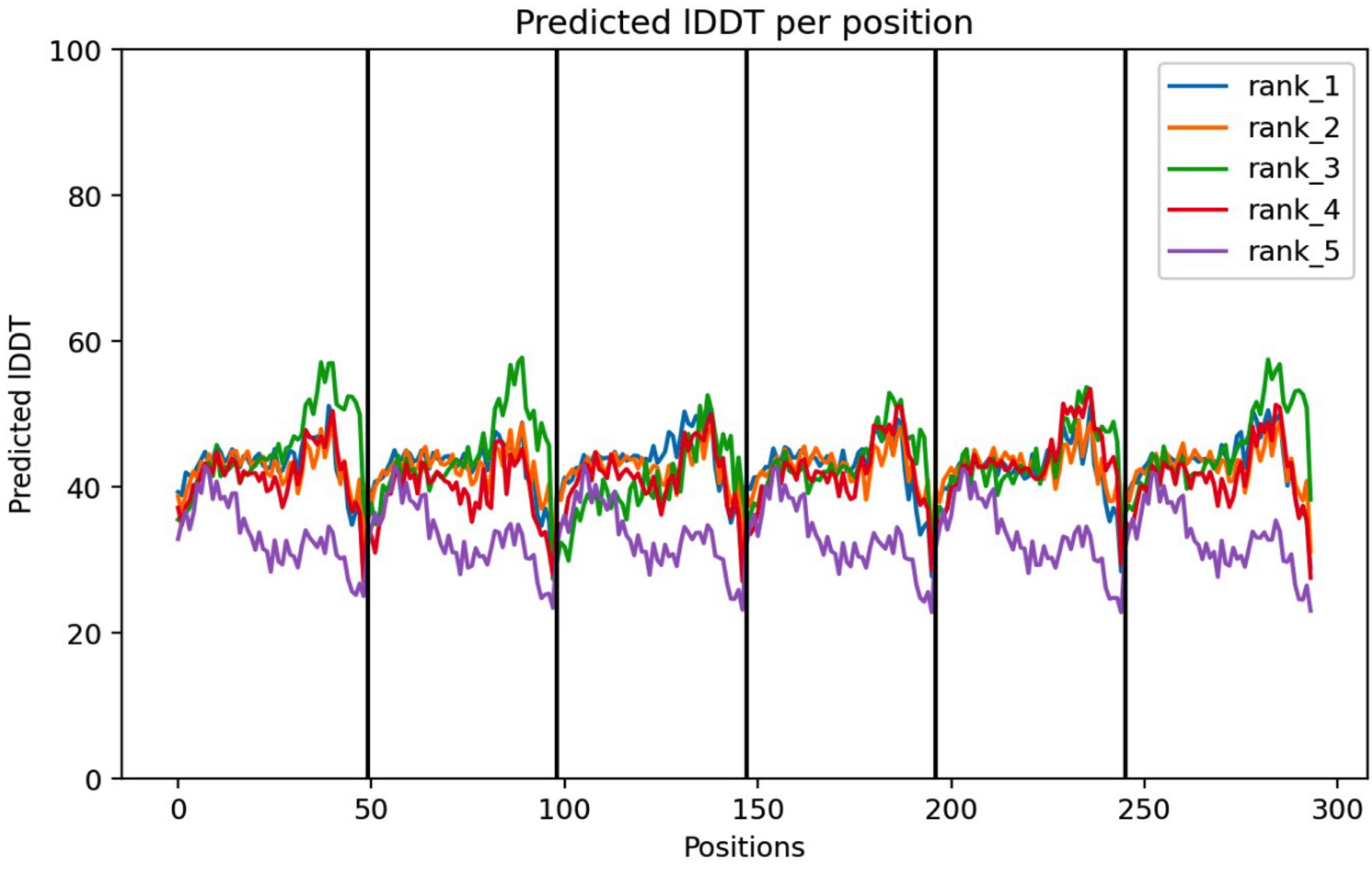

Supplementary Figure S2c.

A15.5L trimer

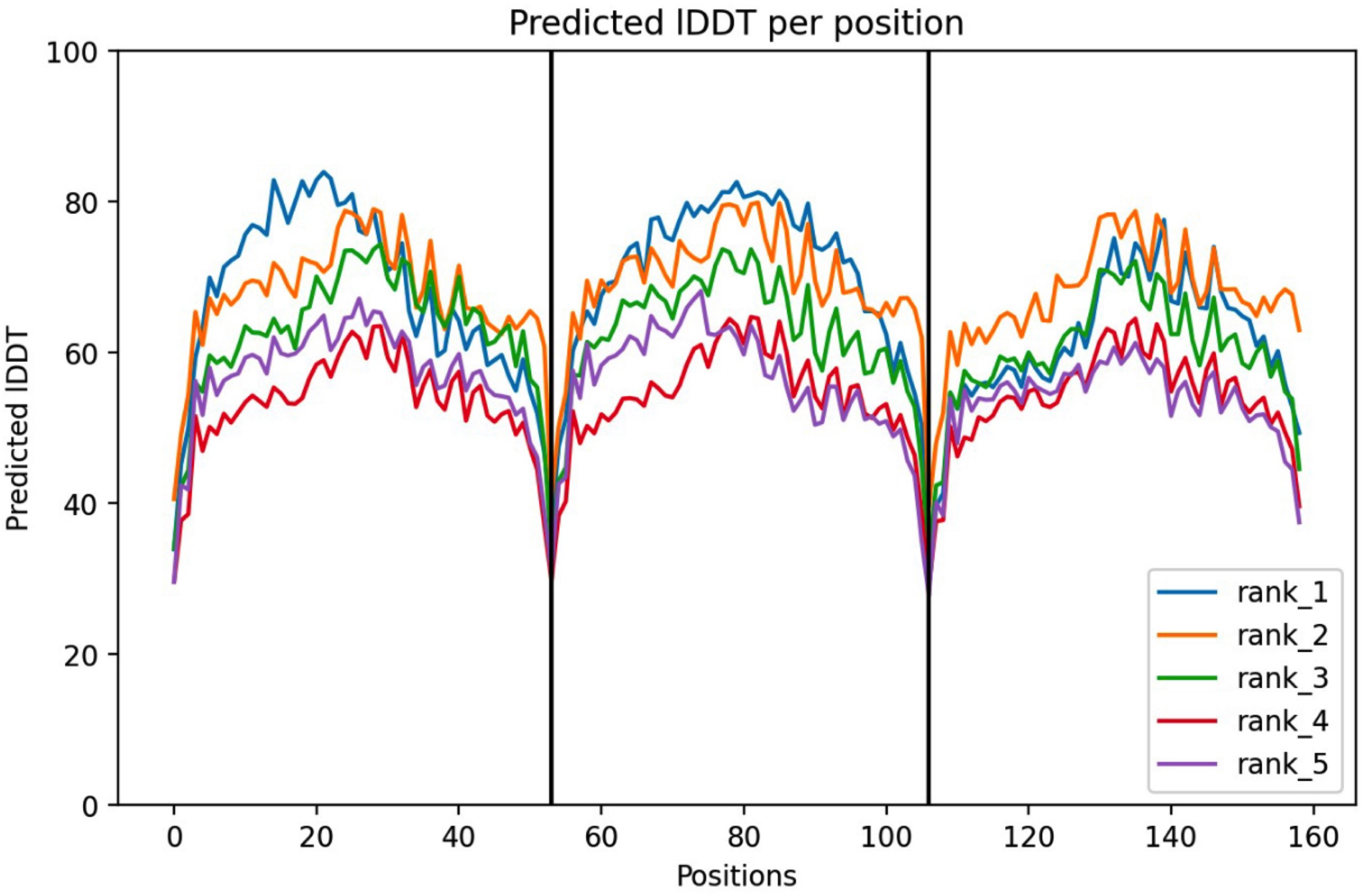

A15.5L tetramer

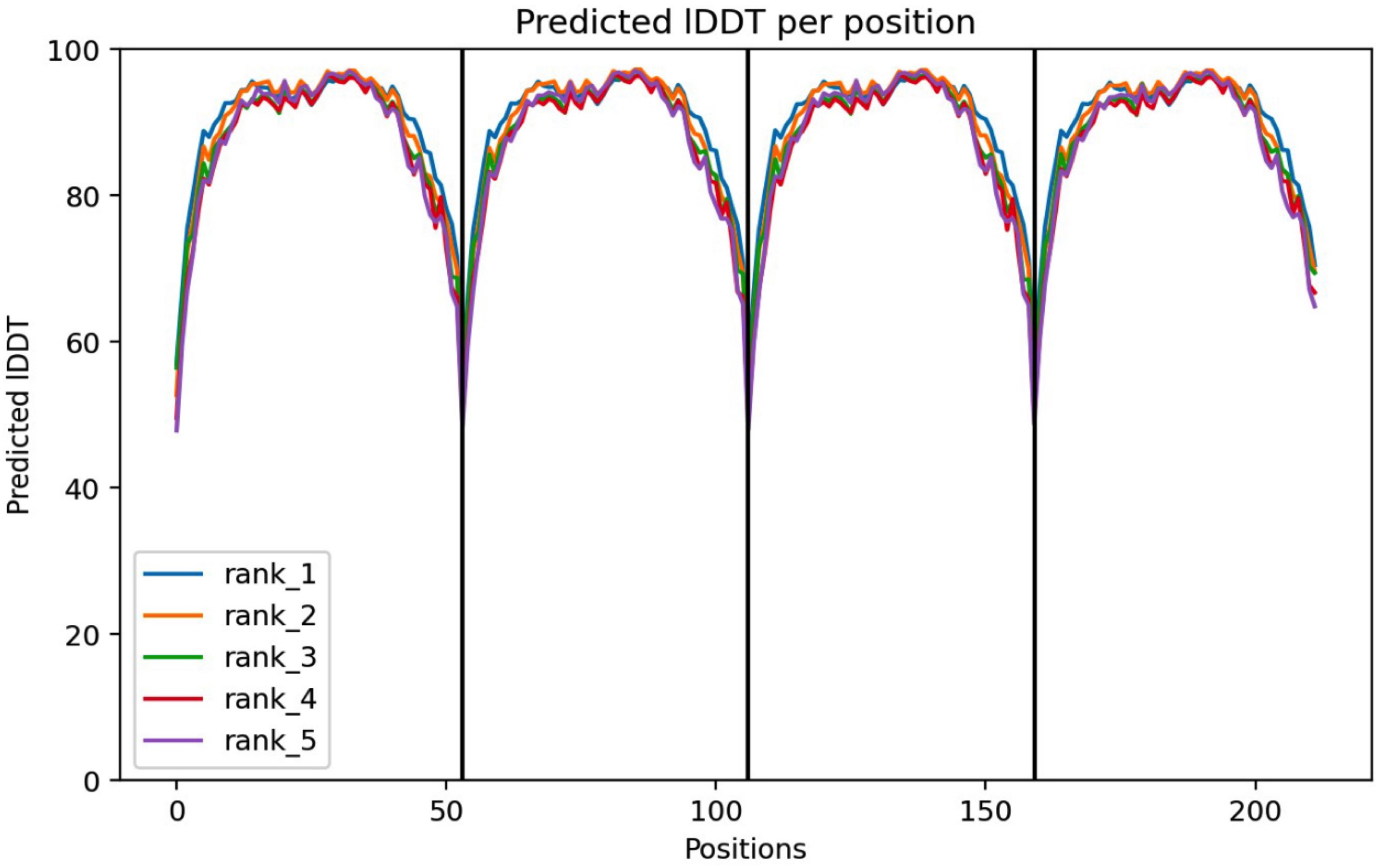

Supplementary Figure S2d.

A15.5L pentamer

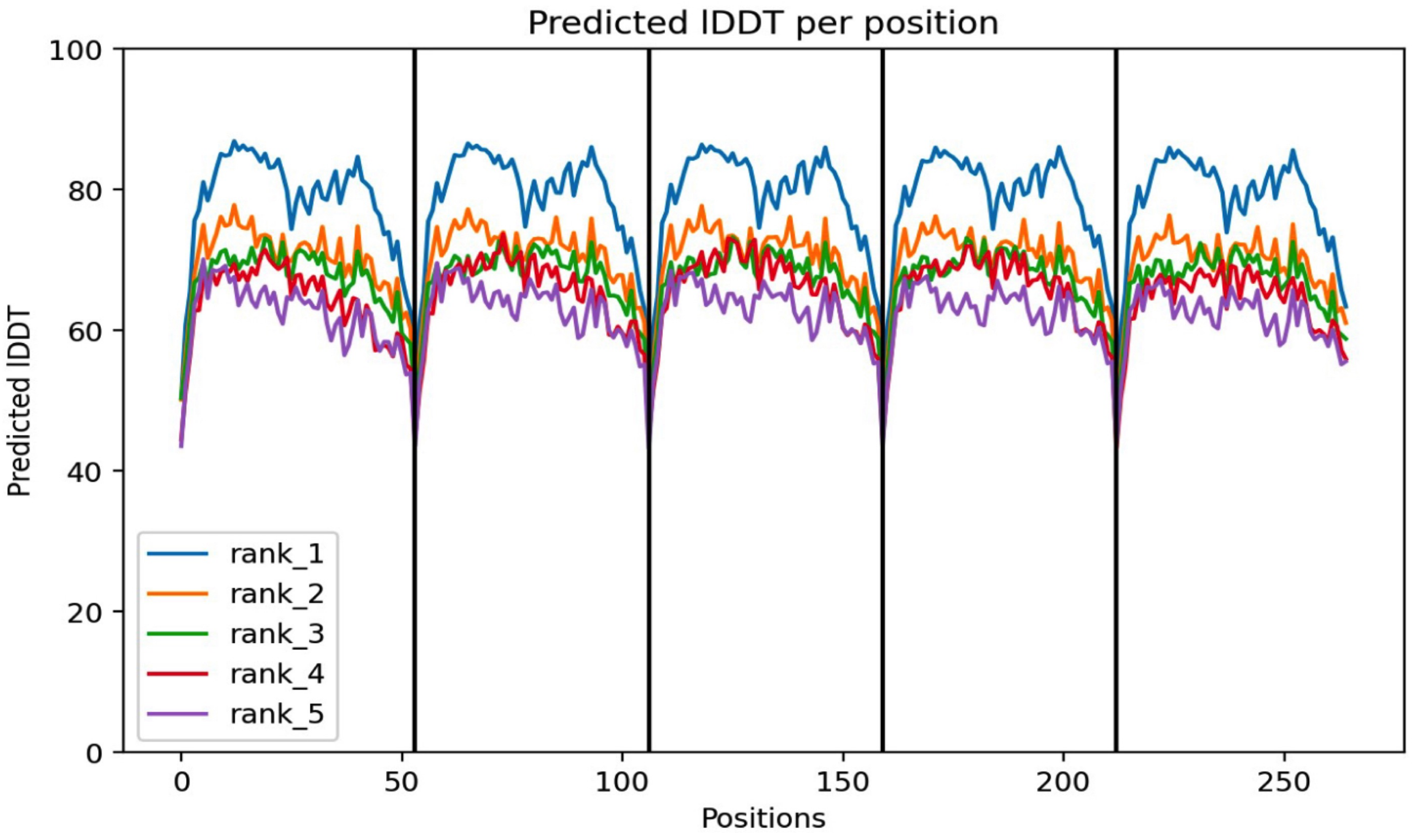

A15.5L hexamer

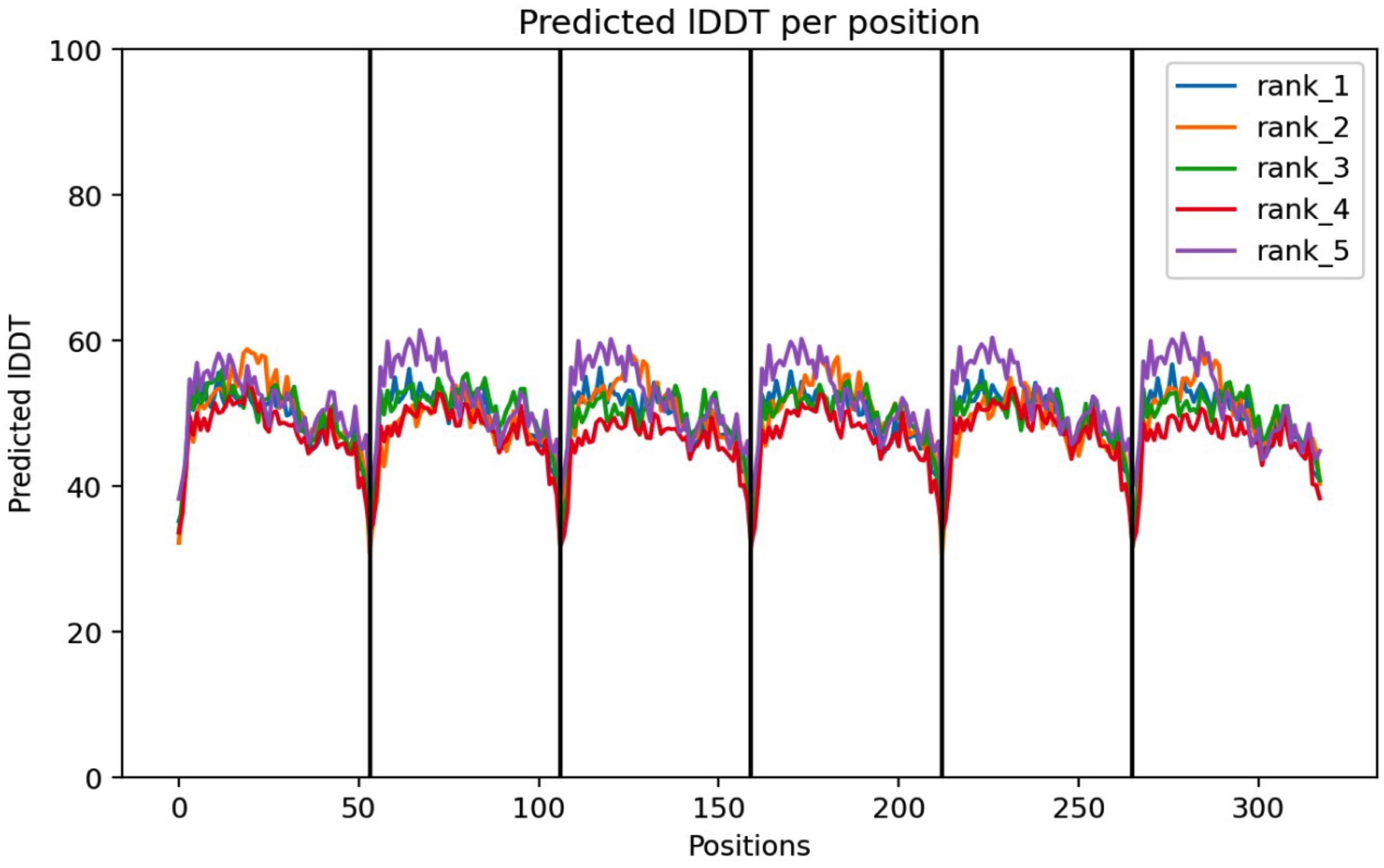

Supplementary Figure S2e.

gp063 trimer

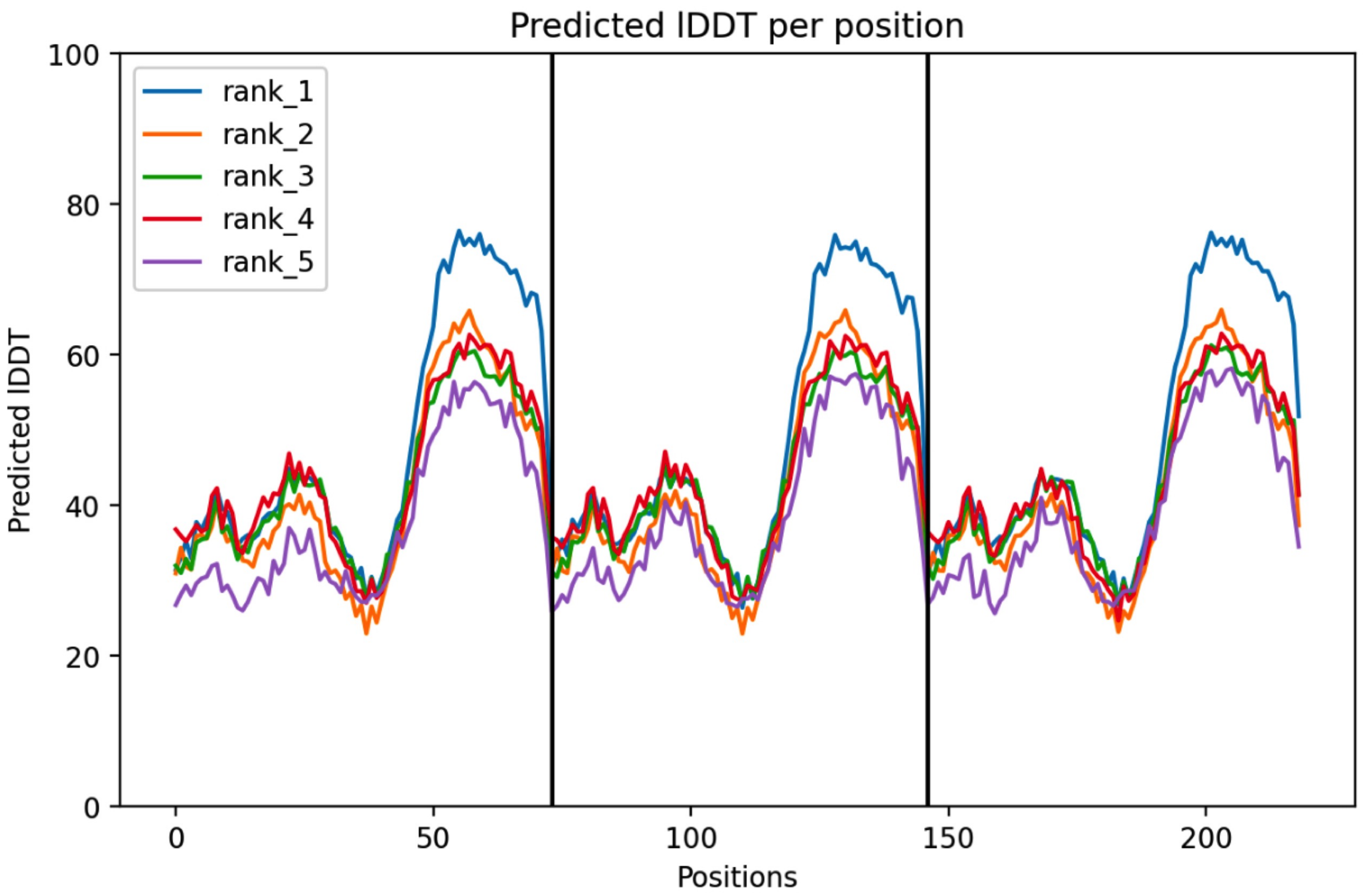

gp063 tetramer

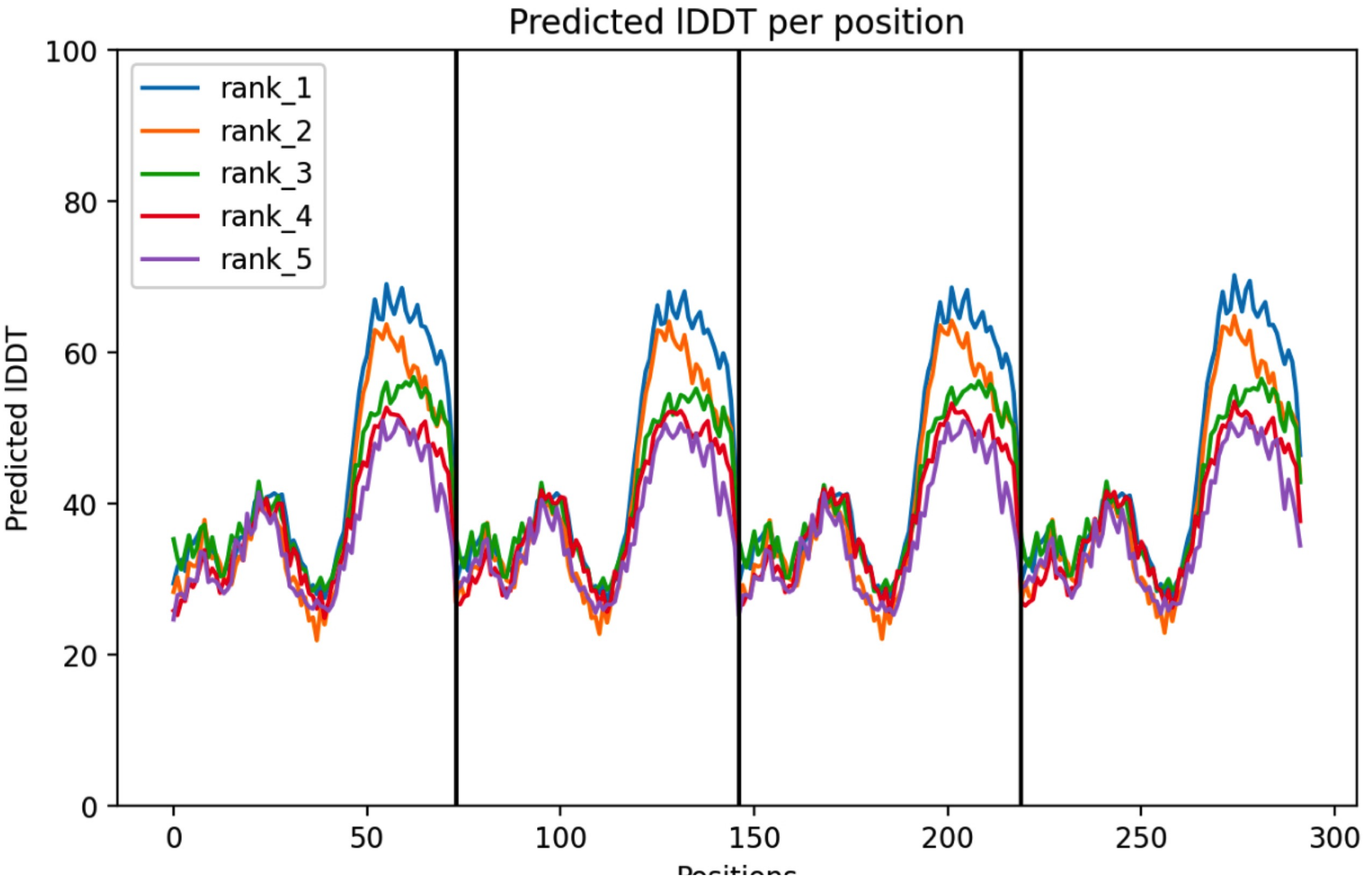

Supplementary Figure S2f.

gp063 pentamer

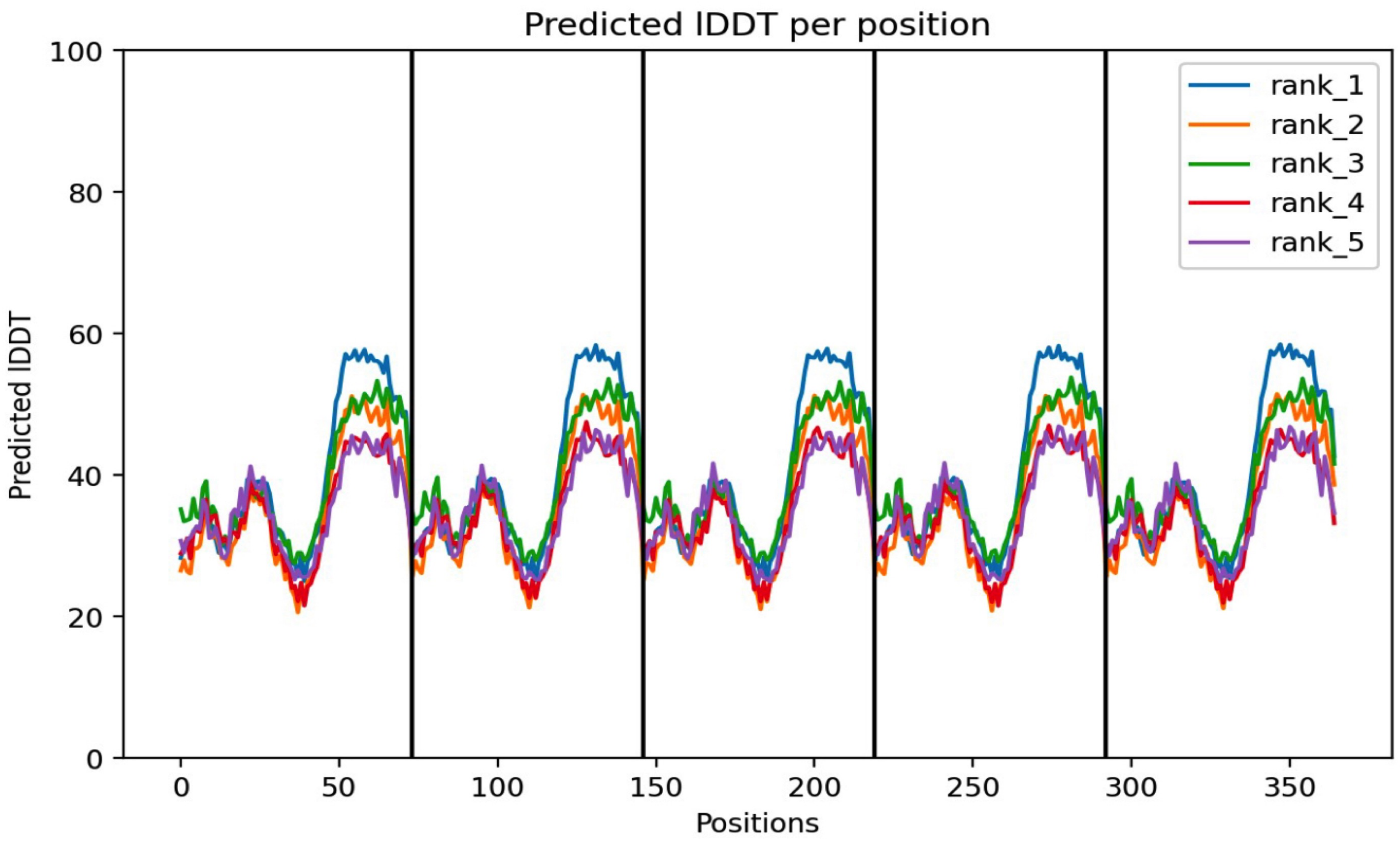

gp063 hexamer

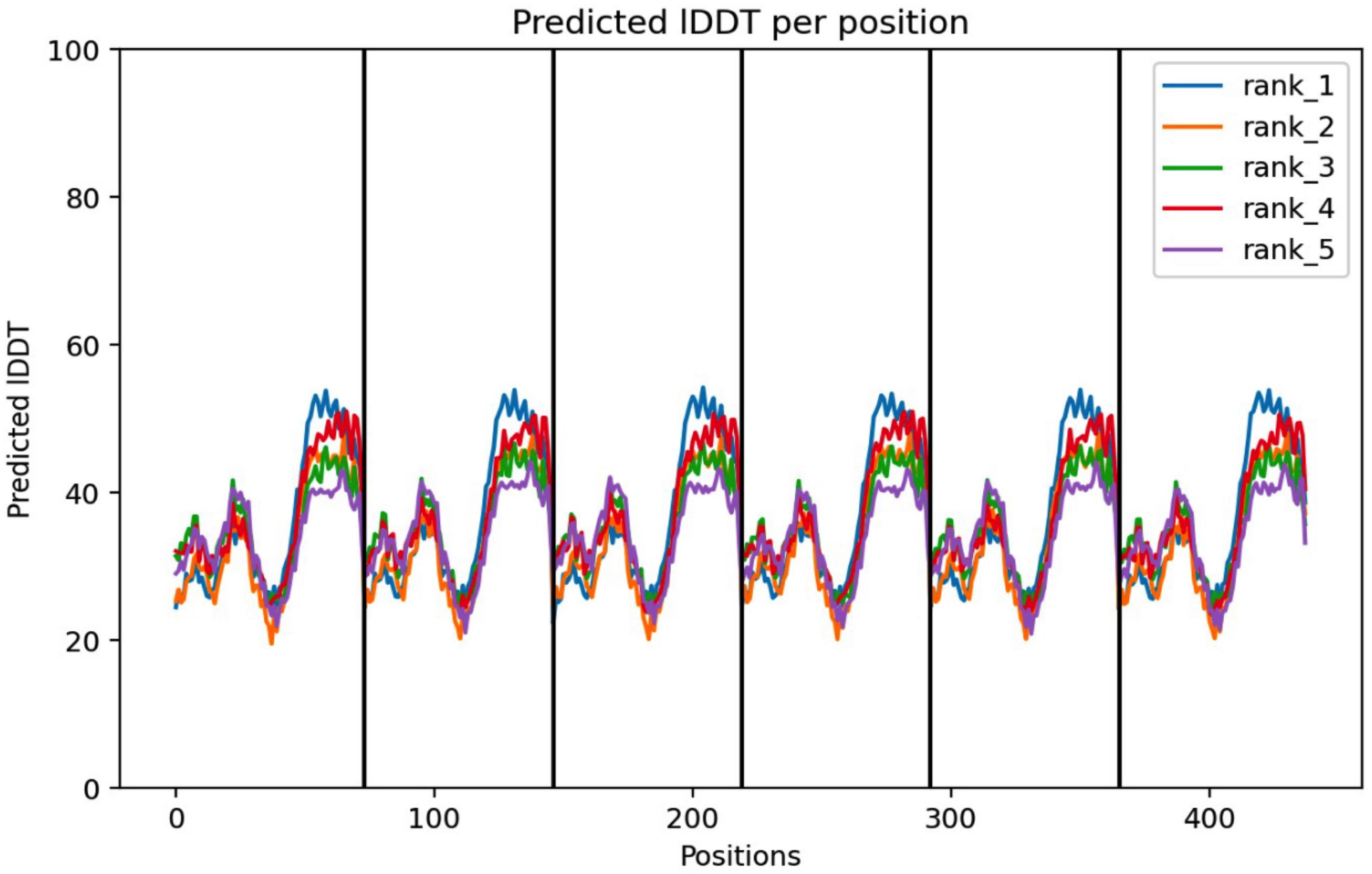

Supplementary Figure S2g.

gp081 trimer

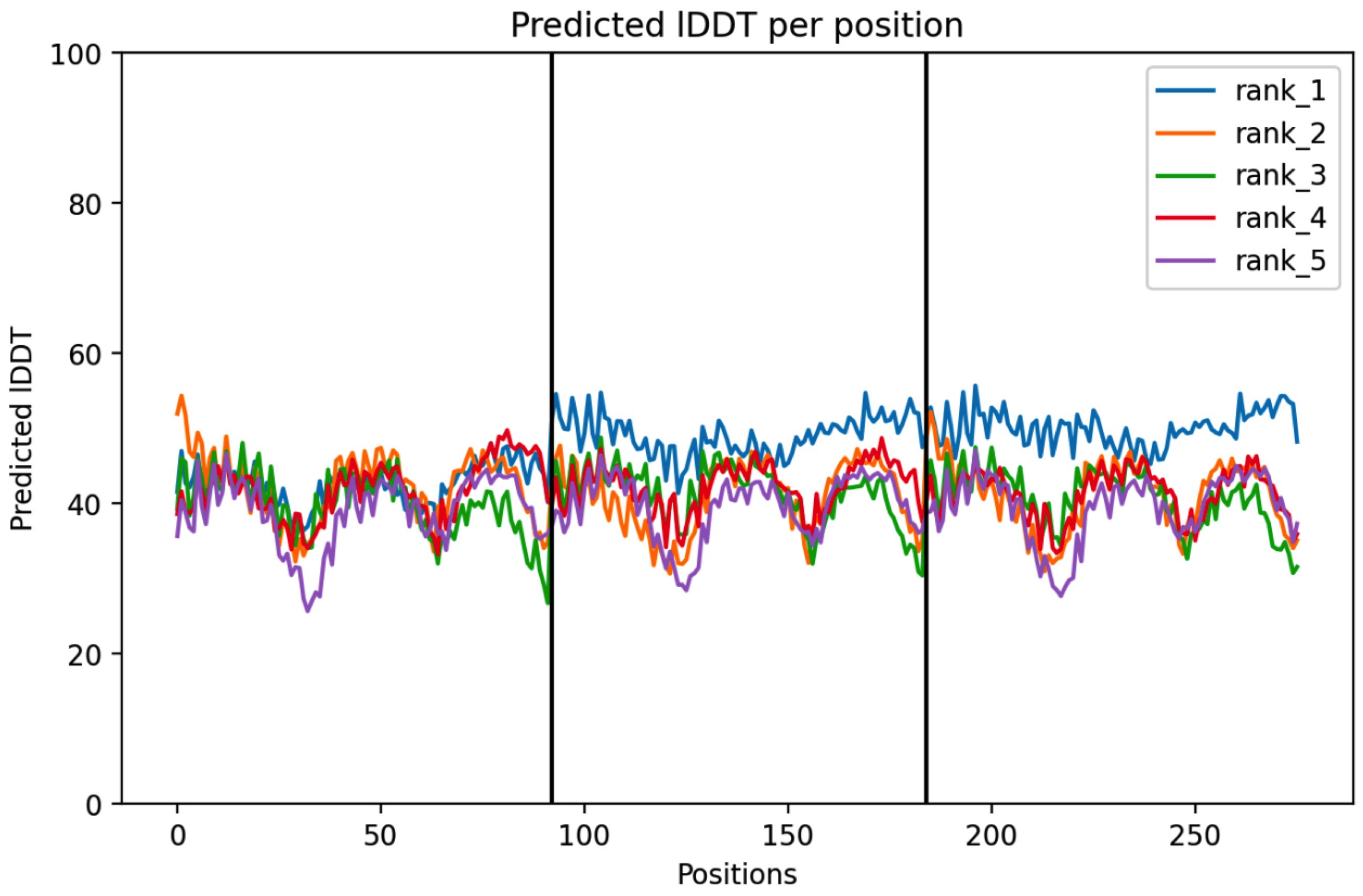

gp081 tetramer

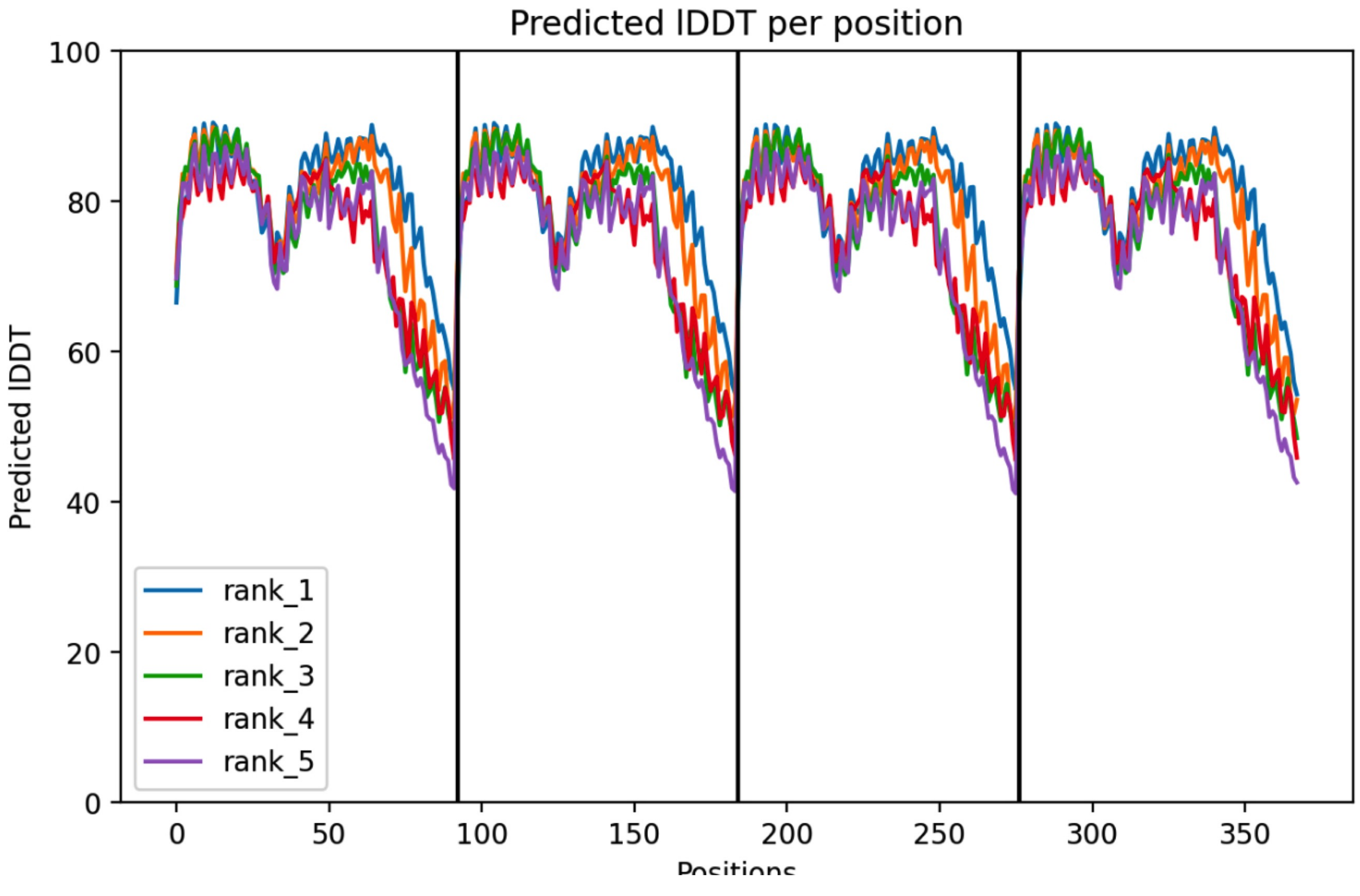

Supplementary Figure S2h.

gp081 pentamer

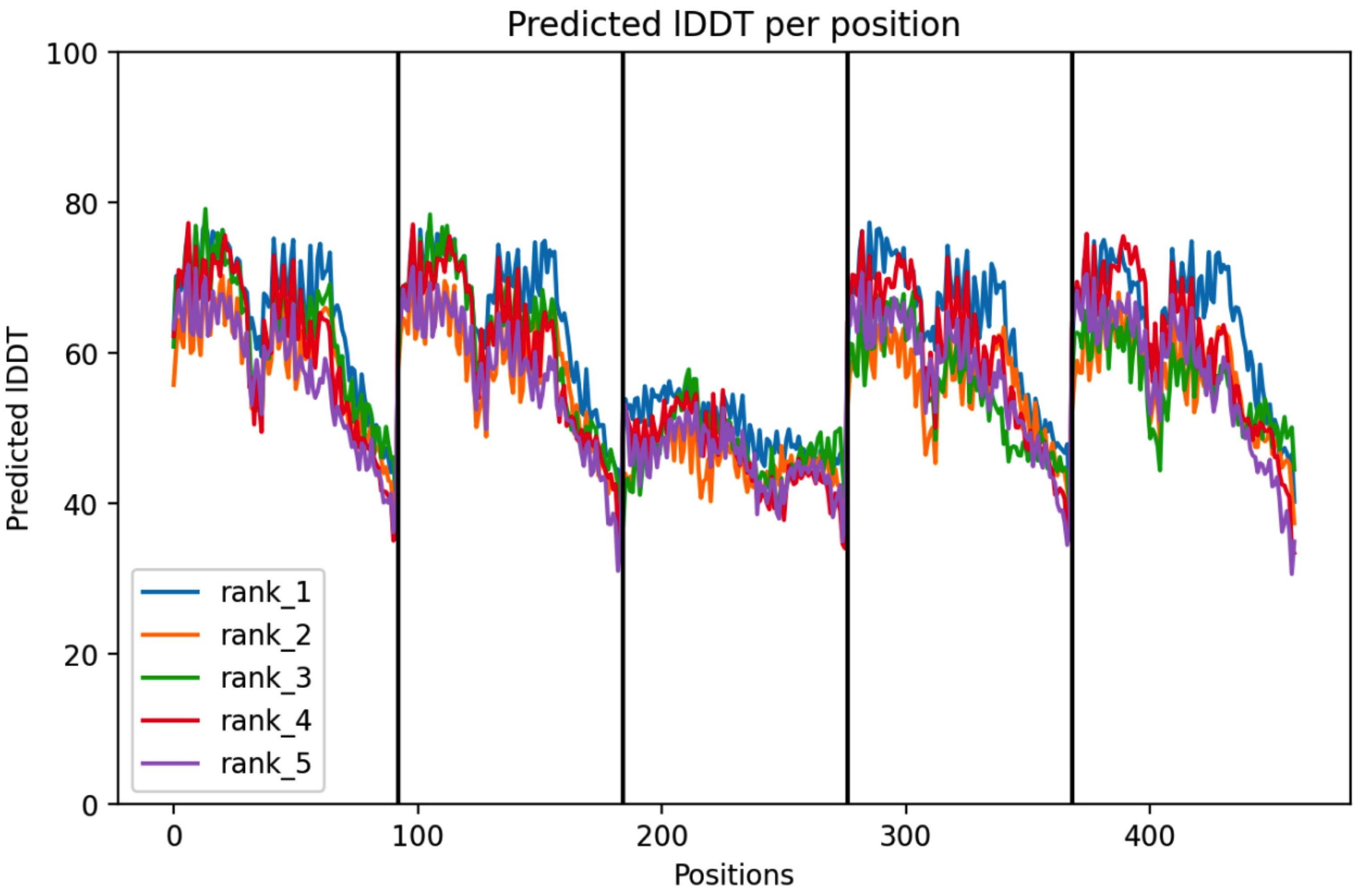

gp081 hexamer

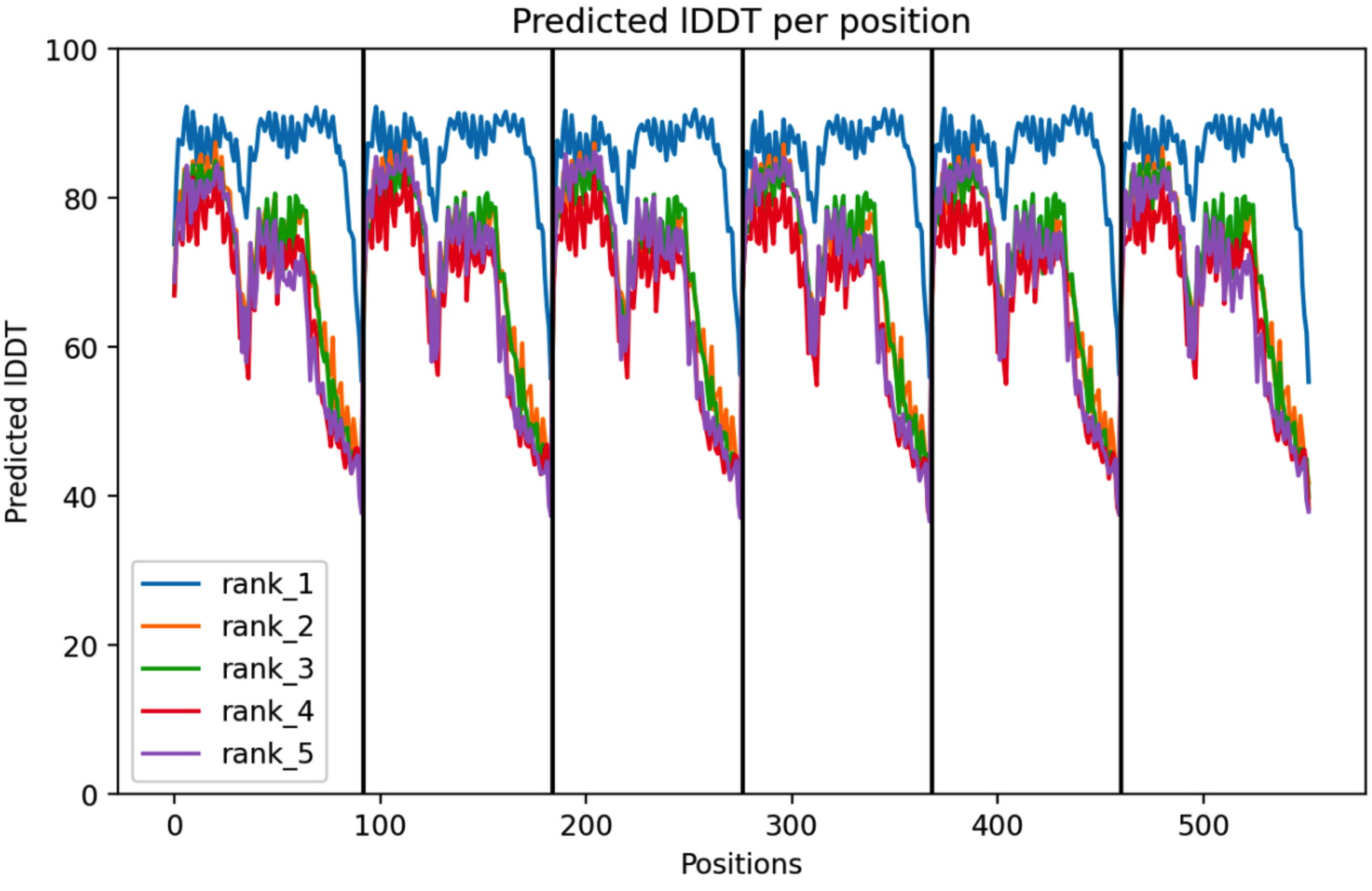

Supplementary Figure S2i.

gp120 trimer

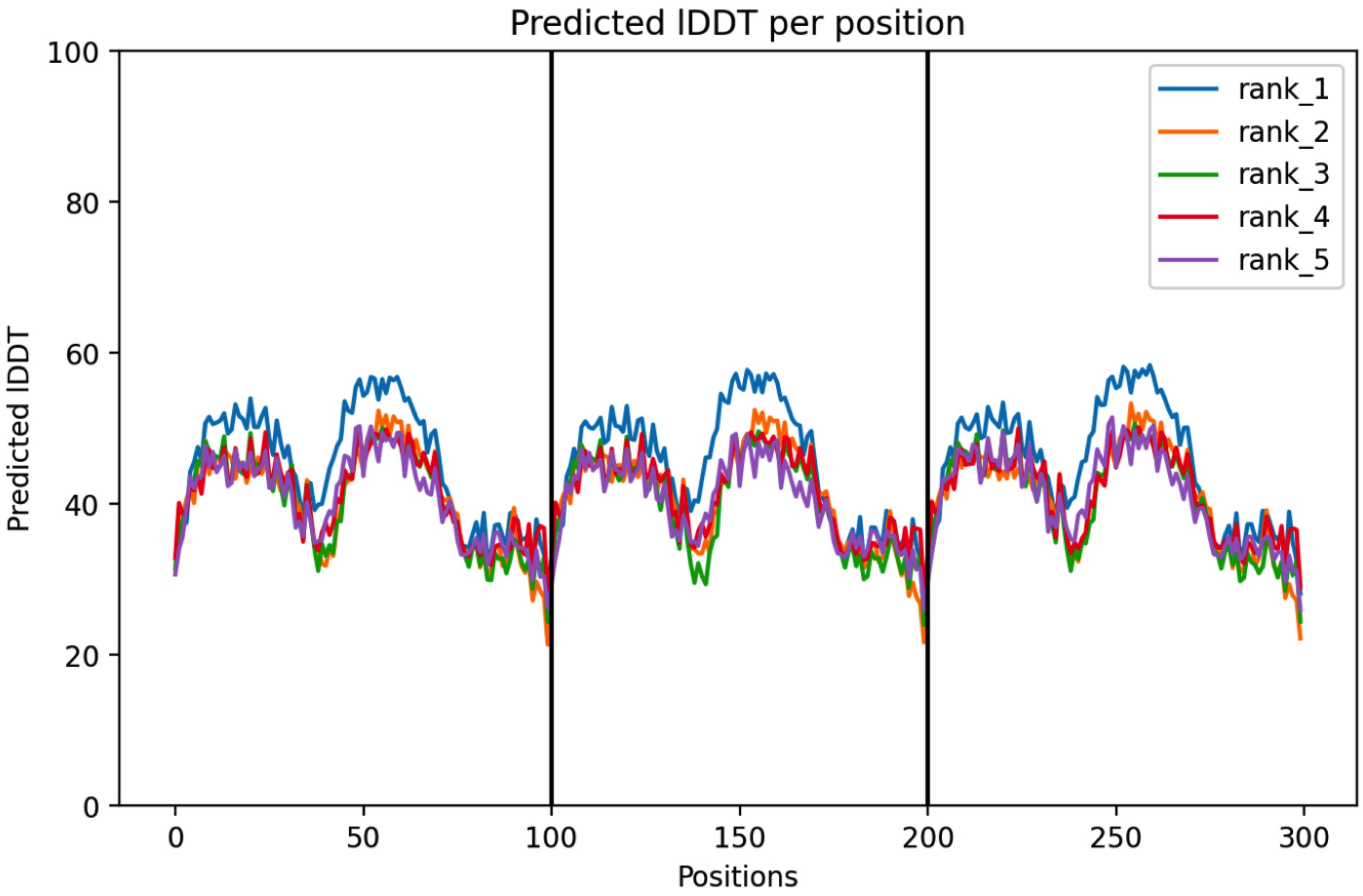

gp120 tetramer

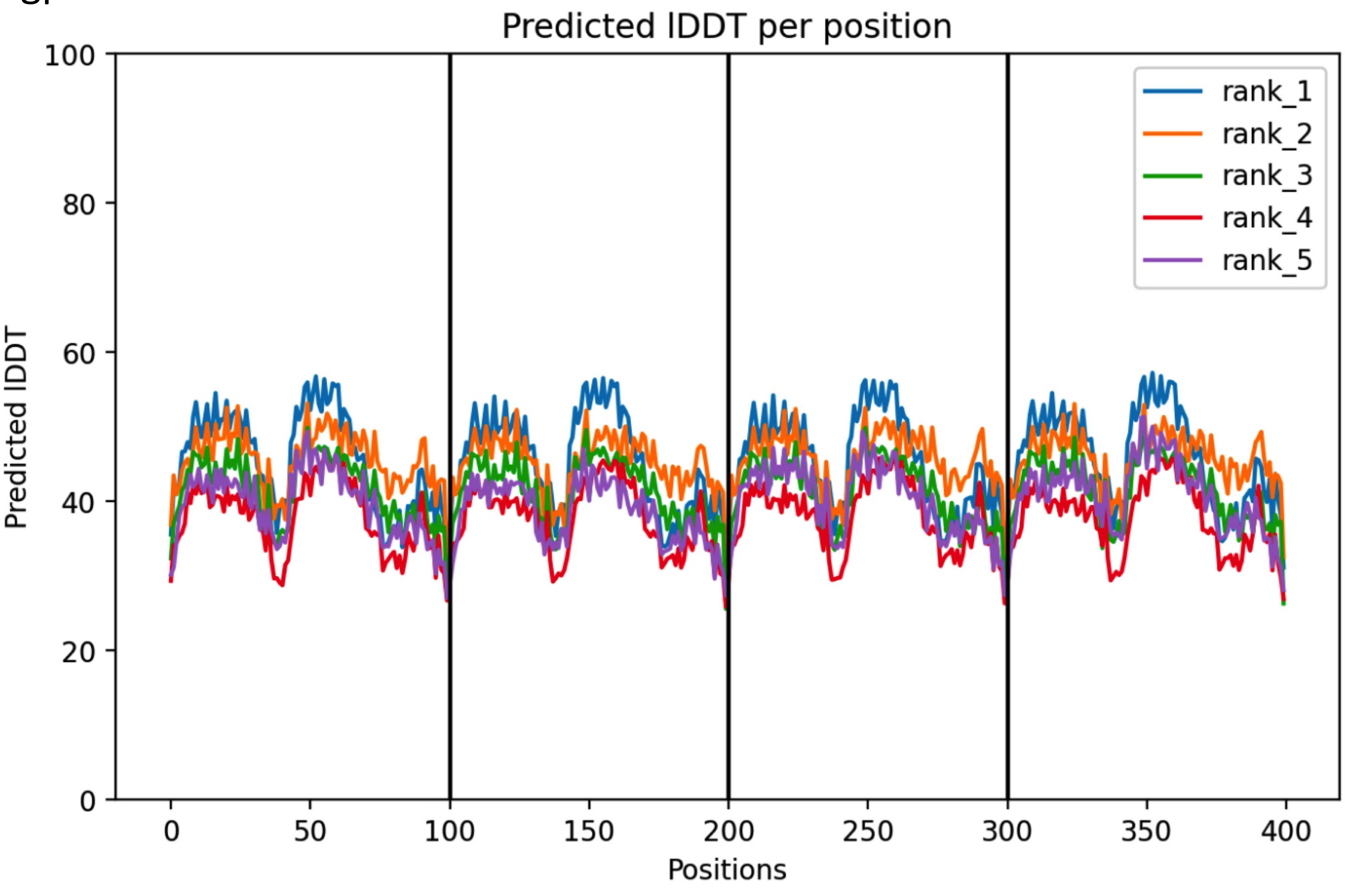

Supplementary Figure S2j.

gp120 pentamer

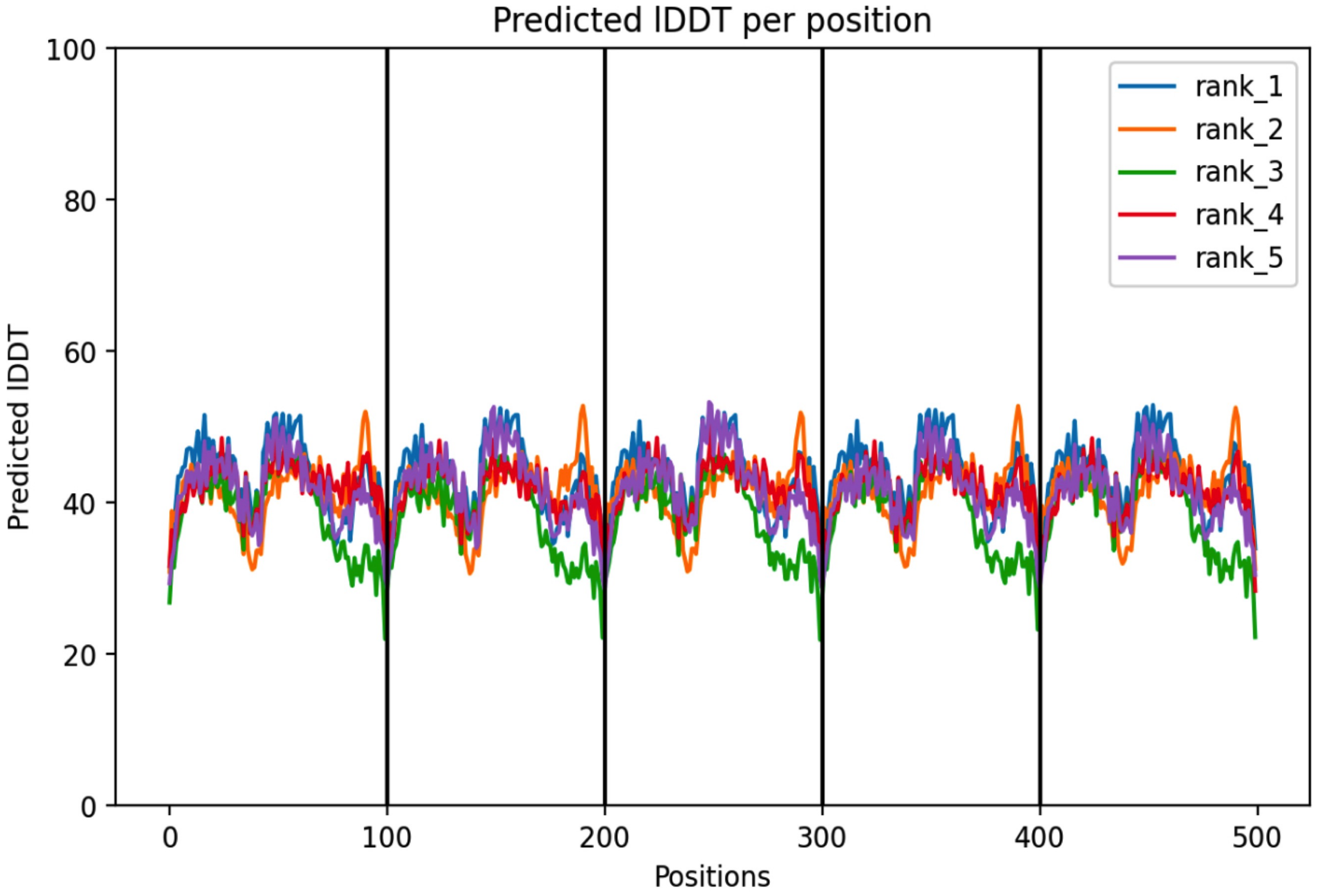

gp120 hexamer

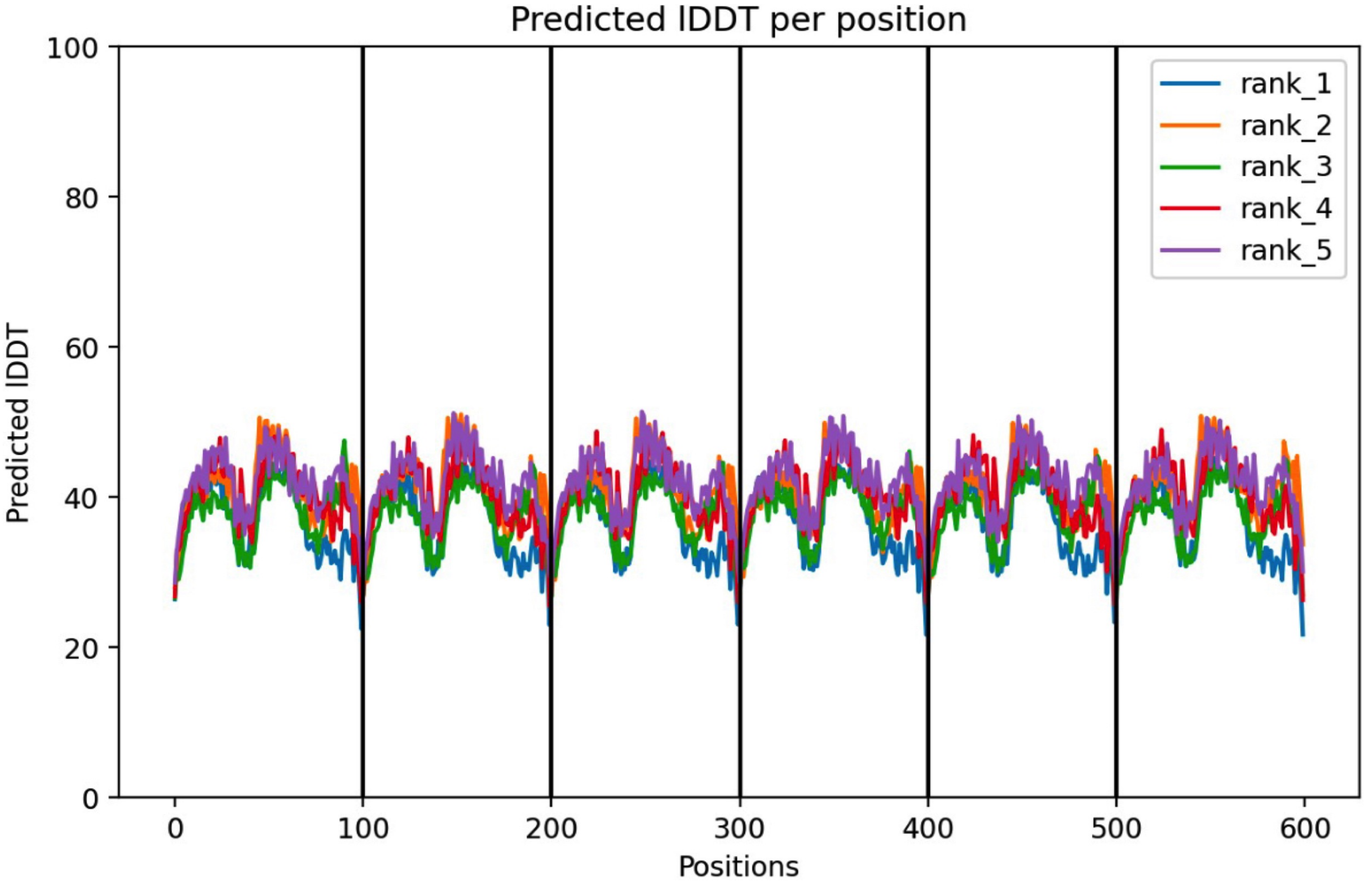

Supplementary Figure S3. AlphaFold2 predicted structures of C20.5L, A15.5L, gp063, and gp120 proteins with panels depicting the confidence level of the corresponding structure with respect to the amino acid positions. Cyan coloring represents the protein surface with polar residues while orange represents hydrophobic amino acids.

C20.5L Supplementary Figure S3a.

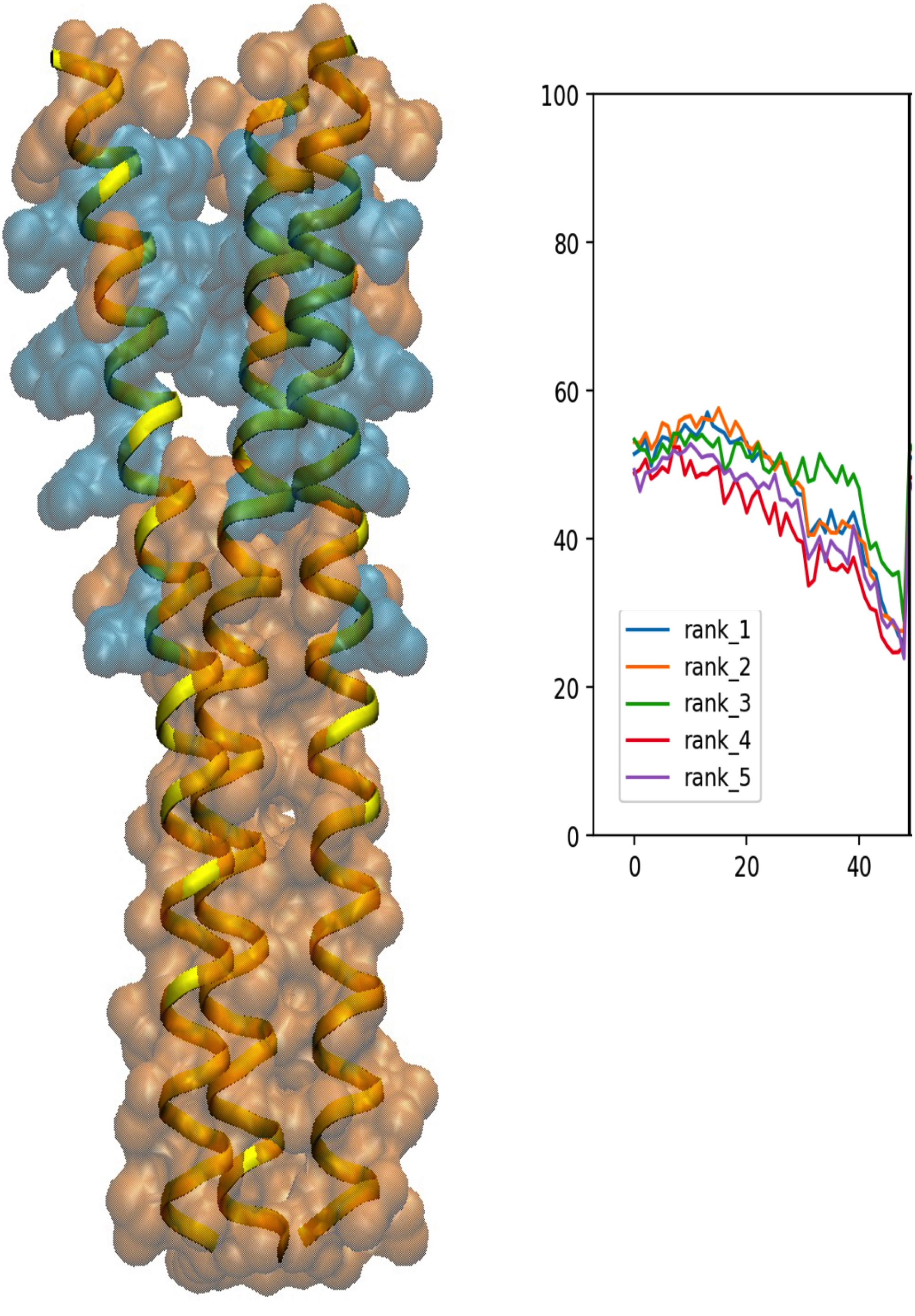

Supplementary Figure S3b.

A15.5L

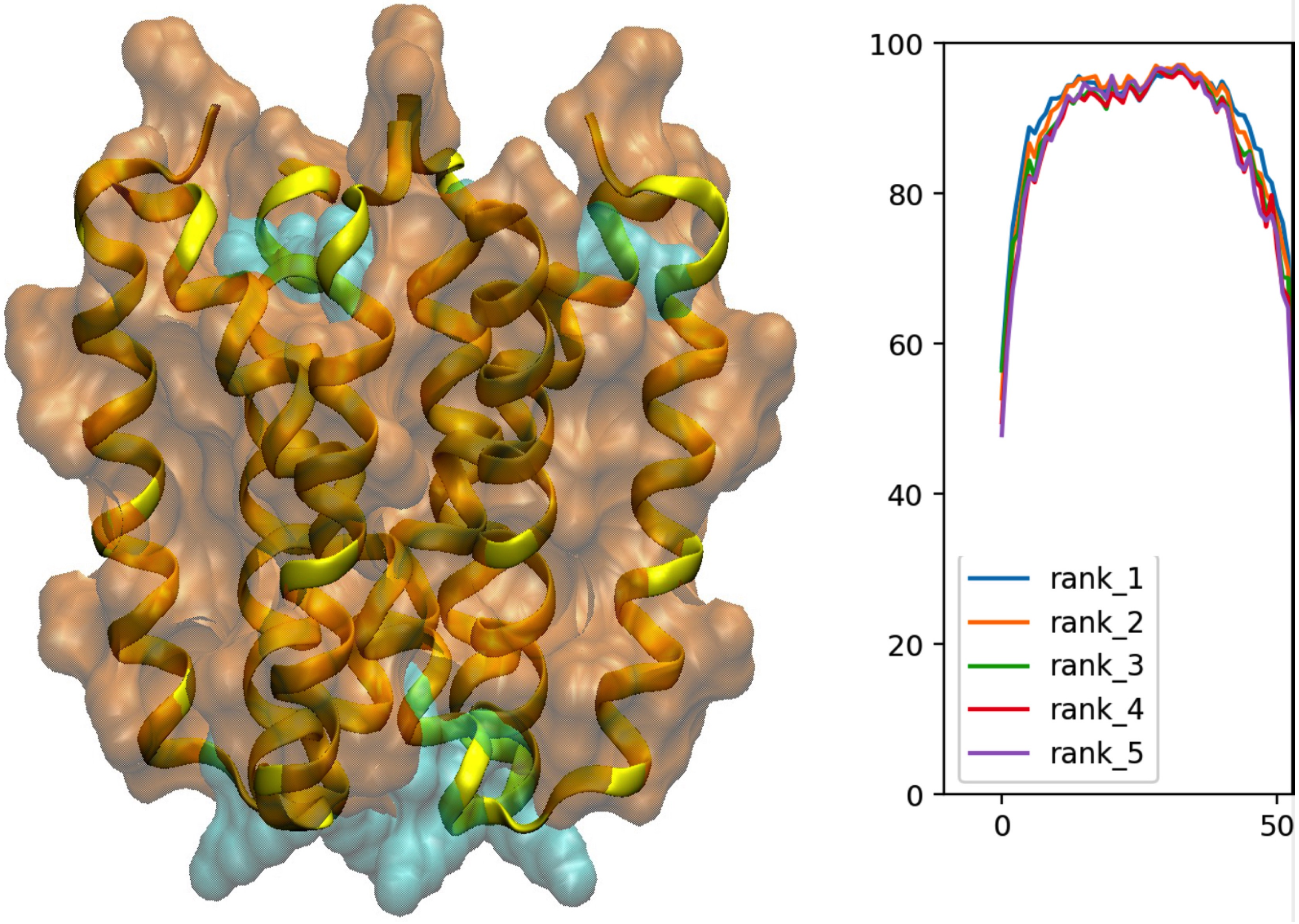

gp063 Supplementary Figure S3c.

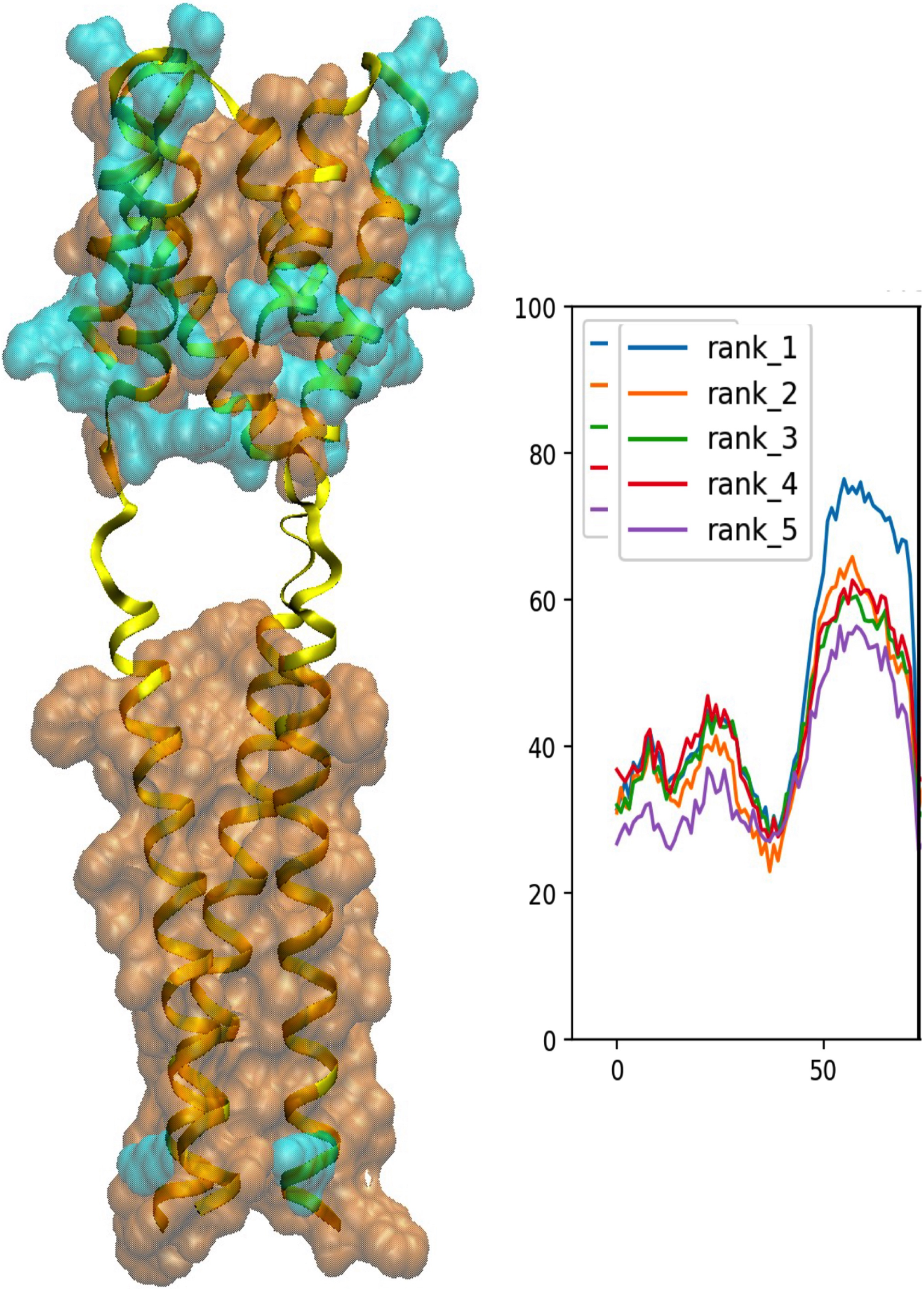

Supplementary Figure S3d.

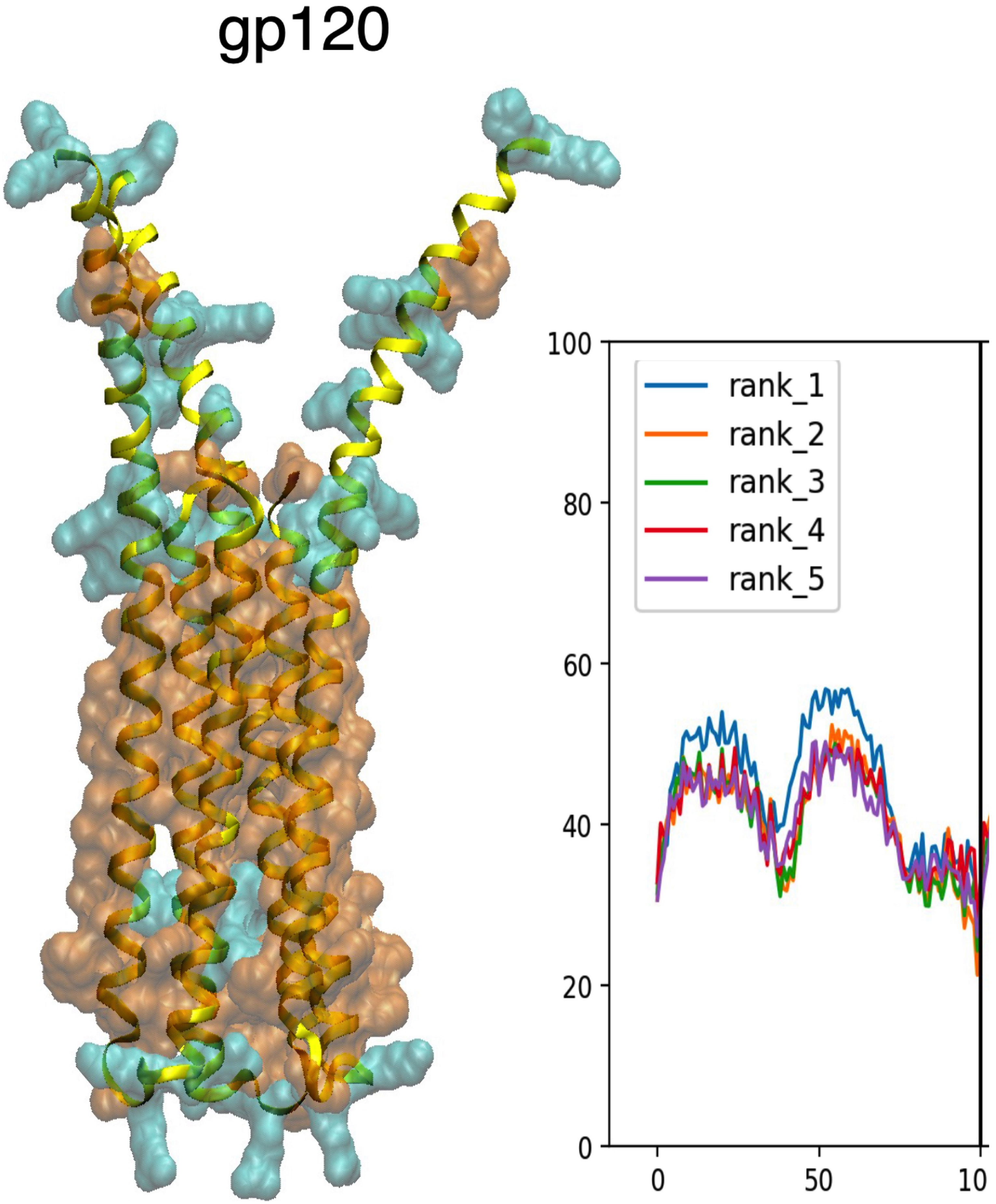

Supplement: Supplementary file 1 [file ijms-24-13828-s001.zip › ijms-2563941-supplementary.pdf]
